# Observations on the Use and Abuse of Mercury in the Cure of the Syphilis

**Published:** 1786

**Authors:** Thomas Kirkland

**Affiliations:** Member of the Royal Medical Society of Edinburgh


### THE
### LONDON MEDICAL JOURNAL,
### FOR THE YEAR 1786,
### PART THE FIRST.

I. *Obſervations on the Uſe and Abuſe of Mercury in the Cure of the Syphilis. Communicated in a Letter to* Samuel Foart Simmons, *M. D. F. R. S. by* Thomas Kirkland, *M. D. Member of the Royal Medical Society of Edinburgh.*

SOME time ſince* you were pleaſed to take notice of ſome hints I have given about ſmall doſes of mercury; but as I find enough has not yet been ſaid upon the ſubject to engage the attention of the faculty, and as the next volume I intend to publiſh ſhortly on medical ſurgery will not admit what I have to ſay about the venereal ulcer, I beg leave to offer to your conſideration the outlines of a practice I have purſued many years, in hope of preventing the injury ſtill daily done to conſtitutions by large doſes of this mineral:

* See Vol. V. page 386.

Truly,

Truly, Sir, the effects I have seen from small doses of divided quicksilver induce me to think, we have been much mistaken in the use of mercury; and that a large quantity is noways necessary in the cure of the syphilis: and I have no doubt that the common practice has been as destructive as the disease for which it has been employed. For though it commonly removes the venereal virus, it often leaves behind it a worse disease, by enervating the whole body, and reducing it to a wretched state of health. Ulcers, which were before curable, are not unfrequently converted into a very sordid, irritable, and incurable state, unless the nerves and fibres can be restored again to their proper strength; for though it is possible to cleanse them, and lessen their irritability, yet if mercury is not laid aside, and the patient's strength regained, they remain inactive, their edges become hollow, the lips of the sore keep dying, and gradually wearing away, and I have not only seen most horrid ulcers, but dropsies, consumptions, &c. and even death, in consequence of this mal-treatment. Surely a worse malady cannot seize the viscera than large quantities of unction bring on through the whole intestinal canal, which, from an apthous or ulcerated ulcerated ſtate, becomes incapable of bearing even the lighteſt food without occaſioning a diarrhœa, in which much ichor is commonly diſcharged, and the patient almoſt conſtantly ſuffers from tormenting gripes; or if he eſcapes this violence, he frequently experiences a pain about the bones, in many parts of the body, that makes his life miſerable, and long fruſtrates the attempts of his aſſiſtant to give relief. Nor will this account appear, to men of experience and obſervation, to be overcharged; for though it has been ſuppoſed by ſome, that untractable ulcers, following a courſe of mercury, are moſtly owing to the ſcurvy, by which I imagine a ſharpneſs of the juices is meant, yet when it is conſidered, that antiſcorbutics do not avail in ſuch caſes, and that when we are ſucceſsful, it is by a treatment that gives vigour to the nerves, and conſequent increaſe of ſtrength to the muſcular and other fibres, it will be pretty evident to what cauſe theſe ailments are owing, eſpecially as they do not happen when a contrary method is purſued.

Inſtead, therefore, of rubbing into the thighs, &c. a load of quickſilver in hog's lard, I have conſtantly for many years given ſmall quantities of it divided in ſtarch. A ſcruple of mercury in in a drachm of ſtarch, made into a maſs with a little water, or thin mucilage of gum Arabic, is divided into twenty pills, two of which are taken daily, excepting thoſe days on which the bowels are opened; and if continued a proper length of time, I believe will ſeldom fail to correct, or remove, the venereal virus in the manner already ſhewn *: but an opening medicine muſt be given once a week, otherwiſe, in ſome habits of body, the mouth, as I have, in a former publication †, obſerved, will be apt to be affected by the time a few grains are taken, unleſs the mercury thus combined proves to be a gentle purge, which is not uncommon; owing, I ſuppoſe, to the acid in the ſtarch occaſioning ſpiculæ, that ſometimes irritate the bowels ‡, becauſe plain quickſilver produces no ſuch effect: but they are much milder in this than in any other mercurial preparation.

I have given quickſilver undivided, in the manner Dovar directs, in aſthmatic caſes; and have freſh inſtances of conſiderable quantities paſſing through the body without producing

* Introduc. to Med. Surgery, p. 107 and 108.

† Med. Surgery, vol. i. p. 497.

‡ See Remarks on Calomel, in the Eſſay on the Child-bed Fever, p. 114.

any any effect; and it is well known, that large quantities of mercury in hog's lard, adminiſtered for the cure of the ſyphilis, often run through the body without any advantage to the patient *. I formerly followed the practice of giving fifteen or twenty grains of quickſilver twice a day in conſerve of roſes; but having diſcovered that calomel was decompoſed in the ſtomach †, I began to reflect on the effects of the very ſmall doſes of mercury in ſome of the mercurial preparations; and from time to time, as experience directed me, I gradually leſſened the quantity of quickſilver, till I diſcovered that the doſe I had preſcribed was more ſpeedy and certain in making a favourable alteration in venereal ſymptoms than any I had before uſed; and the more I ſee of this matter, the more I am inclined to believe what was before ſuggeſted ‡, that giving ſmall doſes of this remedy adds to its efficacy.

Indeed, I have ſo repeatedly and conſtantly ſeen (where large doſes of mercury have not previouſly been employed) venereal ulcers, both in the throat and other parts of the body,

* See Dr. Swediar's work on the Venereal Diſeaſe.

† Eſſay on the Child-bed Fever, p. 114.

‡ Med. Surgery, vol. i.

and other venereal ſymptoms, give way in a ſhort time to this medicine, aſſiſted with proper dreſſings, that I have not any doubt of its ſuperſeding the other antiſyphilic remedies, if perſevered in long enough after the ulcers and other venereal ſymptoms diſappear. I frequently continue it three or four months, with an intention of making ſure work, eſpecially as the patient is never incommoded, or experiences any ſort of trouble, in the proceſs. He may eat and drink as a ſenſible man ought to do. A moderate quantity of good wine is no impediment to the remedy, and, when able, he may work, or uſe recreations, as uſual. Neither need we be alarmed with the falſe appearances which not unfrequently preſent themſelves; for it ſometimes happens, that venereal obſtructions, incapable of being removed by internal remedies, break into ſmall ulcers in different parts of the body after the original complaint is ſubdued, which are only local, and give way to mild dreſſings without requiring internal medicines.

Nor have I confined this method of giving mercury to venereal complaints, having found it equally effectual in all the diſeaſes in which mercury is ſucceſsfully given; but of theſe we

ſhall

ſhall treat hereafter. I only mean in this place to enforce the doctrine before taught, that the more ſimple the preparations of mercury are, the better; that exceedingly ſmall doſes of quickſilver, continued a conſiderable time, are fully ſufficient for every internal purpoſe in which it can be uſeful; and that large doſes are not only unneceſſary, but often violently pernicious. All which will be farther explained in the work before me; though I am perſuaded, if this ſhort abſtract is attended to, the proceſs adviſed will recommend itſelf.

